# Crucial Role of Vitamin D in the Musculoskeletal System

**DOI:** 10.3390/nu8060319

**Published:** 2016-06-01

**Authors:** Elke Wintermeyer, Christoph Ihle, Sabrina Ehnert, Ulrich Stöckle, Gunnar Ochs, Peter de Zwart, Ingo Flesch, Christian Bahrs, Andreas K. Nussler

**Affiliations:** Eberhard Karls Universität Tübingen, BG Trauma Center, Siegfried Weller Institut, Schnarrenbergstr. 95, Tübingen D-72076, Germany; elkewintermeyer@gmx.de (E.W.); Cihle@bgu-tuebingen.de (C.I.); Sabrina.Ehnert@med.uni-tuebingen.de (S.E.); Ustoeckle@bgu-tuebingen.de (U.S.); Gochs@bgu-tuebingen.de (G.O.); PdeZwart@bgu-tuebingen.de (P.d.Z.); Iflesch@bgu-tuebingen.de (I.F.); Cbahrs@bgu-tuebingen.de (C.B.)

**Keywords:** vitamin D, vitamin D deficiency, bone health, muscle pain, chronic disease, supplementation

## Abstract

Vitamin D is well known to exert multiple functions in bone biology, autoimmune diseases, cell growth, inflammation or neuromuscular and other immune functions. It is a fat-soluble vitamin present in many foods. It can be endogenously produced by ultraviolet rays from sunlight when the skin is exposed to initiate vitamin D synthesis. However, since vitamin D is biologically inert when obtained from sun exposure or diet, it must first be activated in human beings before functioning. The kidney and the liver play here a crucial role by hydroxylation of vitamin D to 25-hydroxyvitamin D in the liver and to 1,25-dihydroxyvitamin D in the kidney. In the past decades, it has been proven that vitamin D deficiency is involved in many diseases. Due to vitamin D’s central role in the musculoskeletal system and consequently the strong negative impact on bone health in cases of vitamin D deficiency, our aim was to underline its importance in bone physiology by summarizing recent findings on the correlation of vitamin D status and rickets, osteomalacia, osteopenia, primary and secondary osteoporosis as well as sarcopenia and musculoskeletal pain. While these diseases all positively correlate with a vitamin D deficiency, there is a great controversy regarding the appropriate vitamin D supplementation as both positive and negative effects on bone mineral density, musculoskeletal pain and incidence of falls are reported.

## 1. Introduction

Vitamin D came to the center of attention when it was shown to play a crucial role in many acute and chronic diseases. Based on the ideas of Funk, vitamin D was discovered some 100 years ago and first described by McCollum [1,2,3]. In 1952, it was shown that vitamin D affects the serum calcium and is required for neuromuscular function [1]. Experiments with radioactive ^3^H vitamin D_3_ in the late 1960s assumed that there is a biologically active form of vitamin D which is quite important for the metabolism of organisms [2]. Following this, it took some more years to isolate and name those active metabolites (e.g., 25(OH)D and 1,25(OH)D) [3,4].

According to existing literature, vitamin D deficiency is a global problem with various symptoms and major consequences. It does not only affect the bone quality but also increases the risk of fractures, autoimmune diseases, and cancer [4,5,6,7]. Therefore, a good understanding of the etiology of vitamin D deficiency should be developed to find an appropriate way to treat it.

## 2. Vitamin D—Chemistry, Physiology and Metabolism

Vitamin D is a complex lipophilic molecule whose elemental formula is C_27_H_44_O. There are two sources of delivering vitamin D: (1) cutaneous synthesis and (2) diet.

Most of it is supplied by cutaneous synthesis [4,6,7,8,9]. 7-Dehydrocholesterol (provitamin D_3_, 7-DHC), which is synthesized inside the liver from cholesterol, converts to previtamin D_3_ while absorbing solar energies (ultraviolet B wave length: 290–315 nm). Under the influence of heat, it immediately converts to vitamin D_3_ (cholecalciferol) [7,10]. Although the exposure to direct sunlight, depending on latitude, season and time, makes up the total amount of the vitamin D_3_ production, excessive exposure can also cause the formation of inactive photoproducts [7,9,11,12].

The smaller portion of vitamin D, whereof two forms exist, is ingested from diet [6,7,12] together with calcium and phosphates. Ergocalciferol (vitamin D_2_) is especially found in plants or plant products, while cholecalciferol (vitamin D_3_) is mainly contained in animal products like fresh and canned salmon, mackerel and tuna as well as cod liver oil [6,7,13]. Circulating in the blood, vitamin D and its metabolites are always bound to vitamin D-binding protein (DBP). DBP carries vitamin D to the liver where it is metabolized by the vitamin D-25-hydroxylase enzymes, also known as cytochrome P450 (CYP) 2R1, and CYP27A1. Its product, the biologically inactive form (25-hydroxyvitamin D, 25(OH)D) is recirculated in the blood to be transported to the kidneys for further metabolism. The next step of the vitamin D metabolism is the hydroxylation of 25(OH)D in the kidneys to 1,25(OH)2D (1,25-dihydroxyvitamin D, calcitriol) by the enzyme 25-hydroxyvitamin D-1-hydroxylase (1-OHase), also known as CYP27B1 [3,7]. Accumulation of both 25(OH)D and 1,25(OH)2D can act as a negative feedback mechanism for vitamin D_3_ metabolism, by inhibiting 25-OHase (CYP2R1) and 1–OHase (CYP27B1), respectively. To prevent this, excessive 25(OH)D and 1,25(OH)2D are metabolized by the 25–hydroxyvitamin D_3_-24-hydroxylase (24-OHase or CYP24A1) in order to facilitate biliary excretion of the products (24,25-hydroxyvitamin D/24,25(OH)D or calcitroic acid). Furthermore, lipophilic vitamin D can be effectively stored in fat tissue. The effect of vitamin D on adipocytes has not yet been deciphered [14].

**Figure 1:**
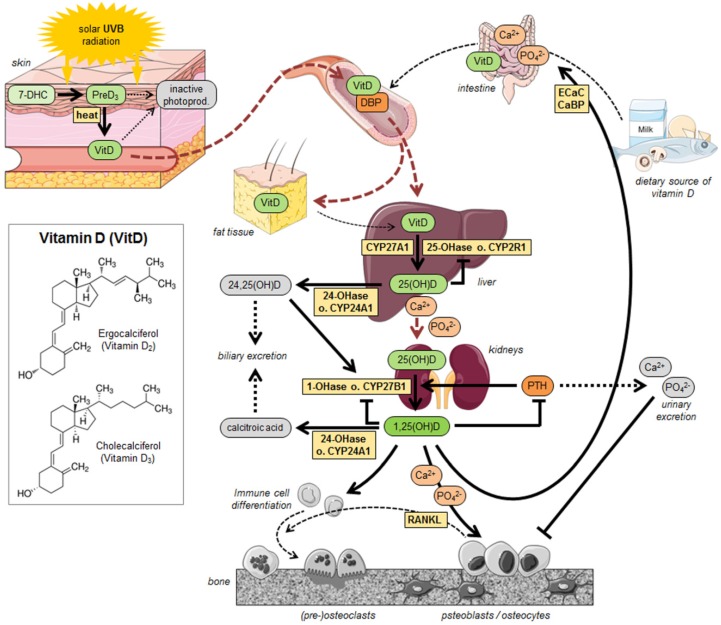
Schematic representation of admission and metabolism of vitamin D. Vitamin D is supplied by cutaneous synthesis or diet intake. The bloodstream takes it into the liver, where its chemical structure is changed by hydroxylation. Then it is sent to the kidneys for another hydroxylation. Finally, the active metabolite 1,25(OH)D circulates through the body in order to be effective. This graphic has been drawn up based on the schematic representation created by Shinchuk 2007 and Heath 2006 [6,7]. © 2016 Laboratoires Servier [22].

Vitamin D metabolism is self-regulated through negative feedback mechanisms, serum phosphate and calcium, fibroblast growth factors (FGF-23), and parathyroid hormone (PTH) (Figure 1) [4,15]. The overall reduction of 25(OH)D causes an increase in PTH levels by a negative regulation mechanism: By binding to the parathyroid vitamin D receptor, active vitamin D inhibits PTH gene transcription [16]. This, in turn, may increase bone turnover and consequently bone loss by increasing bone FGF-23 gene expression [17,18]. FGF-23, in turn, might enhance vitamin D inactivation both positively and negatively by 1a-hydroxylase and 24-hydroxylation of 25(OH)D [19]. This probably explains why increased PTH levels are negatively correlated with bone mineral densiy (BMD) in patients with secondary osteoporosis [20,21].

By influencing the PTH concentration, estrogen, glucocorticoids, calcitonin and somatotropin can also affect the calcitriol synthesis. As 1,25(OH)D increases the catabolism of itself by enhancing the expression of the enzyme 24-OHase (CYP24A1), it also directly inhibits the anabolic enzyme 1-OHase (CYP27B1) or indirectly by inhibiting the production of PTH in the parathyroid glands [3,4,15].

Furthermore, 1,25(OH)D regulates the phosphate and calcium balance by supporting the intestinal absorption via epithelial calcium channels (ECaC) and calcium–binding proteins (CaBP). To obtain its biological activity, 1,25(OH)D needs to be bound to the vitamin D receptor (VDR) whose property it is to form heterodimers with related receptors (e.g., the retinoid X receptor, (RXR)) and bind to vitamin D response elements (VDREs) to initiate intracellular signaling cascades [4,12,15,23,24]. As one possible response, 1,25(OH)D enhances the expression of the receptor activator of NFKB ligand (RANKL) in osteoblasts. RANKL associates with the receptor activator of NFKB (RANK) on immune cells to induce differentiation into osteoclasts to release calcium and phosphorus from the skeleton into the blood [4,7]. Nevertheless, active vitamin D compounds have been successfully used as therapeutic treatment for osteoporosis. Paradoxically, in these patients, an increase in bone mineral density is obtained resulting from an inhibition of the osteoclastogenesis [25].

It isn’t noteworthy that, the non–classical actions of vitamin D, e.g., regulation of the renin–angiotensin system, may play a relevant role in the mortality and morbidity of patients with secondary osteoporosis [26,27]. This cascade leads to a sequential activation of angiotensin II, which is likely to have deleterious effects on blood pressure and the vasculature. Thus, decreased 25(OH)D levels are thought to predict hepatic and renal decompensation in these patients [28,29].

## 3. Vitamin D—Prevalence of Deficiency and Insufficiency

Vitamin D deficiency is a widespread and global problem. To determine vitamin D status, 25(OH)D is measured in the blood [4,7,10,30,31]. Insufficient vitamin D levels are at 20 ng/mL to 29 ng/mL, levels below 20 ng/mL are defined a deficiency [4,6,7,9,32]. There is recent evidence that 25(OH)D levels underlie a circadian rhythm, therefore, special care has to be taken during blood sampling [33].

Between 2001 and 2006, about a third of the US population was clearly affected [10,34]. A German health interview and examination survey for adults showed that slightly more than 60% of the German population have 25(OH)D levels below 20 ng/mL [35]. Amling and Barvencik reported an investigation showing that in 2012, about 80% of the European inhabitants had a vitamin D serum concentration below 30 ng/mL [13,36]. The probability of suffering from vitamin D deficiency increases with age [32].

Vitamin D insufficiency and deficiency can occur in various ways. Causal to the deficiency can be, among other things, the reduced intake with diet, inadequate sun exposure or a metabolic disorder [6,7,8,9]. Risk factors to developing vitamin D insufficiency or deficiency are: (a) reduced or restricted sun exposure (e.g., homebound persons, people covering their skin for cultural/religious reasons); (b) a reduced cutaneous synthesis (e.g., in elderly); (c) suffering from a malabsorption syndrome (e.g., Crohn’s disease); and/or (d) obesity [6,7,9,11]. There is no generalized recommendation for the time of sun exposure needed to obtain sufficient vitamin D activation as this process is strongly dependent on skin pigmentation, area of exposure and UV strength [37]. Furthermore, vitamin D is fat soluble and gets easily accumulated in fat–tissue [7,9]. What must not be neglected is the drug-induced decrease of vitamin D (e.g., glucocorticoids) [7].

Regarding the cultural aspect of vitamin D deficiency, abnormalities in vitamin D metabolism in Muslim women were reported several times [38]. The effect of vitamin D deficiency on bone turnover in Muslim women was described by Diamond *et al.* in 2002 [39]. Using urinary deoxypyridinoline (DPYD) as a non–invasive marker of bone resorption, they could show a 5.5-times greater risk of developing high bone turnover if severe vitamin D deficiency was evident. In this cohort of healthy Muslim women, a severe vitamin D deficiency of 68% and a high bone turnover of 46% were shown. Bone densitometry and fracture risk were not analyzed. Fadoua Allali *et al.* described the impact of clothing style on bone mineral density among post–menopausal women in Morocco [40]. They showed that a clothing style covering arms, legs and head increased the risk of osteoporosis by 2.2-fold. As described by Metcalfe *et al.*, sunlight is a necessary component of vitamin D synthesis and people exposed less frequently to the sun are at increased risk, including women dressed according to the Shariah law. Therefore, poor exposure to sunlight was stated as an extraskeletal risk factor for hip fractures [41].

The optimum setting of 25(OH) blood level is achieved when the serum level is above 30 ng/mL [36]. In 1997, the recommended daily allowance for vitamin D intake to prevent a deficiency was 5 μg (200 IU) from childhood onwards up to late adulthood (50 years of age), 10 μg (400 IU) for people aged 50–70 years, and 15 μg (600 IU) for those over 70 years of age [6]. In presence of an osteomalacia, the weekly intake recommended by current guidelines should be much higher (20,000 to 40,000 IU/week) [42]. The necessary daily doses of vitamin D to prevent falls have been reported to be between 700 and 1000 IU [43]. According to the Swiss guidelines “Medizinische Leitlinien für Diagnostik und Therapie”, the needed loading dose in case of vitamin D deficiency is calculated as follows: (75-measured 25(OH)D blood level) × 40 × kg body weight. Afterwards, a daily intake of 1000 IU is recommended [44]. The supplementation regime is pretty similar to the German one, in which a daily dose of 800–1000 IU in patients with a high risk of falls is recommended [45]. However, as reported in recent findings, there is no further benefit of a vitamin D supplementation beyond the compensation of the deficiency. On the contrary, it might even lead to an adverse outcome [46]. The lowest effective and the optimal dose of vitamin D supplementation can vary with the author, the type of patients and the underlying disease. However, more research still needs to be done to clearly define and optimize the recommendations, since there is recent evidence that the success of vitamin D therapy might even be strongly gender dependent, especially regarding adverse effects on the cardiovascular system [47].

## 4. Bone Health

Vitamin D deficiency is associated with diseases affecting bone health (e.g., rickets, osteomalacia and osteoporosis) [9,30,48]. Ephesus once wrote about a child whose spine curved while sitting upright and whose thigh bones bent under the weight of its body, but never named a disease. At the end of the 16th century, Reusner described a disease occurring in the general population of Switzerland and Holland with features such as torsioned bones. Half a century later, a disease called “rickets” was first named as a reason for death in England [48]. In the same century, descriptions of rickets and its features were published by Whistler (1645), Boot (1649), Glisson (1650) and Mayow (1668) [48,49]. Until well into the 20th century, symptoms of rickets were common in the population of British and other northern European industrial cities with a high prevalence during the winter months [4,48,49,50]. Symptoms of rickets include changes in bone (e.g., deformities of the leg), a swelling of the wrist with a widened growth gap, a delayed closure of the fontanelles, craniofacial dysmorphy and musculoskeletal pain. Further symptoms like a cardiac arrest, a tetany or seizures might be induced by the resultant hypocalcaemia [8]. Depending on its manifestation, rickets can lead to a hospitalization or even death. As of the early 20th century, rickets and its treatment was more thoroughly understood and the number of affected patients decreased [48,49]. Hospitalization rates because of rickets have always been rising and falling in England [51]. However, a constant rise of incidences over these past two decades could be observed [49]. Goldacre *et al.* illustrate that the rate is now at its highest point in the last 50 years [51]. 

Osteomalacia, another metabolic bone disease mainly caused by malfunction of the vitamin D or phosphate metabolism, leads to a reduced bone mineralization in adults [12,42]. Unlike rickets, osteomalacia is rare in children [8]. Reuss-Borst described an association between histological modifications in bone mineralization and the 25(OH)D serum level and states that there are no typical findings in blood levels over 30 ng/mL [12,36]. Heath indicates that osteomalacia is clinically apparent and found in patients with serum levels <25 ng/mL [6]. However, there is no specific blood parameter to prove the presence of osteomalacia. Hence, osteomalacia can be diagnosed on the basis of decreased serum calcium or phosphate levels and an elevated alkaline phosphatase [12,42,52]. On clinical examination, unspecific symptoms like musculoskeletal pain, usually located in the pelvis, the shoulders or the proximal part of the muscles, can be found. Based on those symptoms, diseases with comparable symptoms such as rheumatic diseases must be excluded with the help of differential diagnosis [12,42]. Shinchuk, Reuss-Borst and Rader report that pain increased by mild pressure on the sternum or anterior tibial bone are typical or suspected symptoms [7,12,42]. In radiographic images showing Looser’s zones, a decrease of bone mineral density and increased uptake in bone scintigraphy are typical [12,42,52]. 

In their case report, Lopresti *et al.* demonstrate how difficult and time consuming the diagnostics of tumor–induced osteomalacia can be [53]. They report a patient presenting diffuse costal and vertebral pain without any trauma over a three–year period. Only after performing a scintigraphy showing the increased tracer uptake in osteomalacia’s predestined regions of the skeleton, could the diagnosis could be made. Furthermore, they stressed that a total surgical removal of the neoplasia is always required. 

**Figure 2:**
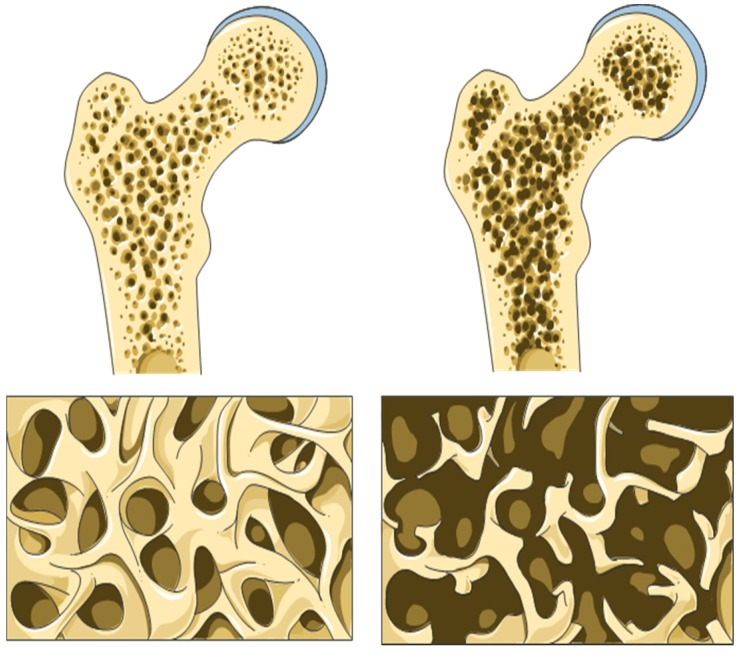
Left: Schematic structure of a healthy bone; dense and loadable bone structure. Right: Schematic structure of an osteoporotic bone; decreased bone mass and pathological changes in microarchitecture. © 2016 Laboratoires Servier [62].

Osteoporosis, a skeletal disease, is characterized by a decrease in bone mass and pathological changes of the microarchitecture due to a low serum level of 25(OH)D, leading to an elevated risk of osteoporotic fractures (Figure 2) [42,54,55,56]. According to the findings of Shane *et al.*, the existence of two single nucleotide polymorphisms (rs4355801 on chromosome 8 and rs3736228 on chromosome 11) constitutes a genetic predisposition which is associated with low bone mineral density (BMD) and a resulting increased risk of fractures [57]. The evaluation of the US National Health and Nutrition Examination Survey III (NHANES III) showed that calcium intake can also affect BMD depending on the 25(OH)D level. Women with low vitamin D levels (<50 nM 25(OH)D) showed a significant increase in BMD when given calcium. Among men, the positive effect remained absent [43]. Jackson and colleagues came to similar results when analyzing the results from the Women’s Health Initiative [58]. The diagnosis of osteoporosis is confirmed by measuring bone mineral density in the lumbar spine or femoral neck by dual-energy X-ray absorptiometry (DXA) [54,57]. In case of osteopenia, the T-score is between −2.5 and −1. In cases of osteoporosis, the T–score is ≤−2.5 [13,55,56]. This is exactly where Siris *et al.* deal out criticism, because having a BMD between ≤−2.5 and ≤−1 and having suffered from a low–energy trauma on a typical osteoporotic fracture site is still classified as having osteopenia [55]. Particularly affected fracture sites include the pelvis, vertebrae, proximal femur/hip, proximal humerus and the forearm [54,55,56,59,60,61].

The telephone health survey by the Robert Koch Institute (RKI) in 2003 revealed that the lifetime prevalence for osteoporosis in women (aged >45) is over 14% and is even rising with increasing age [61]. Wacker and Holick depict a prevalence of about 30% in women aged between 60 and 70 years [4]. Per year, there are approximately 9 million osteoporosis-induced fractures globally (~2 million in the USA), among which fractures of the hip and forearm are most common [30,55,59,63]. The lifetime prevalence of an osteoporotic hip fracture among North American women is about 18% [56]. Johnell outlines a 25% increase in hip fractures between 1990 and 2000 [59]. Bischoff-Ferrari and colleagues expect a further increase of 240% (women aged >65 years) or 310% (men aged >65 years) by the year 2050 [64]. In addition to strong pain, osteoporosis involves specific risks, including a high morbidity and mortality [56,59]. A pooled analysis, describing the effect of vitamin D supplementation on fracture reduction, showed that there was a significant reduction in the incidence of hip fractures when given higher doses than 792 IU/day [64]. However, there is no significant decrease of the hip fracture risk among women and men caused by calcium intake as shown in the meta-analysis of prospective cohort studies. A meta-analysis of randomized, controlled trials adversely showed a significant effect among postmenopausal women with a significantly increased hip fracture risk [65]. The effect of calcium intake on fracture risk still needs to be more thoroughly investigated.

## 5. Vitamin D and Sarcopenia

Over the past decade, the role of vitamin D in sarcopenia, a reduction in skeletal muscle mass and strength due to degenerative processes, has been increasingly reported. Vitamin D affects muscle strength, muscle size and neuromuscular performance [6,11,32]. Evidence suggests that with increasing age, the reduction of muscle mass is clearly associated with decreased circulating vitamin D levels, leading to frailty in the elderly and frequent falls [66,67,68,69]. In order to achieve an effect on the skeletal muscle mediated via the genomic pathway, vitamin D needs to be bound to the VDR receptor [4,12,15,23,24,70]. This is in line with Campbell and co-workers who reported that a decline of specific vitamin D receptors on muscle cells are directly associated with increasing age and the loss of muscle mass and function [71]. Fighting a vitamin D deficit with the help of supplementation has been reported to be beneficial in many studies [66,71]. This is in agreement with the fact that patients with higher 25(OH)D levels have better muscle performances of the lower extremities than patients with lower serum levels [23,43]. The maximum health benefit with regard to sports is reported to be at a level of 50 ng/mL. Higher serum levels do not lead to further benefit [32]. Furthermore, thanks to vitamin D’s support of muscle strength, patients with higher 25(OH)D levels have an increased postural control and a decreased number of falls [5,11,43,72]. A systematic research, including double-blind randomized controlled studies, in which daily doses of 700 IU vitamin D supplementation were administered, showed that falls in elderly people can be reduced if serum levels are higher than 60 nmol/L [43,63]. However, others deny a reduced risk of falling with a once–daily supplementation of 600 IU of vitamin D [32]. This conclusion is in line with a more recent finding suggesting that very high doses of vitamin D are indeed effective in compensating a deficit. However, there is no benefit regarding lower extremity function and, against all expectations, very high doses of vitamin D were even linked with increased risk of falls [73]. This apparent contradiction suggests that vitamin D supplementation must be closely monitored and will need further studies to reach a common treatment regimen. This is particularly important in all kinds of rehabilitation settings where vitamin D deficiency is frequently compensated by diet in order to improve bone and muscle strength. 

Nevertheless, several trials also point out that there is an association between the blood serum level of 25(OH)D and musculoskeletal pain as well as non–specific back pain [6,10,12,23,42,43,63]. However, there seem to be logical contradictions in the published literature [74]. With the help of a muscle biopsy in patients with a vitamin D deficiency, Ceglia characterized atrophy in type II muscle fibers as (1) an enlargement of the interfibrillar gaps; (2) a fibrosis of the surrounding tissue; and (3) an infiltration with fat cells and glycogen granules [23,32]. Heath and Knutsen showed that there is an increase in type IIa fibers (count and diameter) after proper vitamin D supplementation which leads to improved muscle performance [6,11]. 

Gendelmann et al reported findings in literature that describe a nerval hypersensibility caused by a 25(OH)D deficiency [5]. Moreover, they mention a reported estimated 20% prevalence of widespread pain. A cross-sectional study in Norway, including 572 patients with musculoskeletal pain, headaches or fatigue, concluded that more than half of all participants (58%) showed 25(OH)D levels <50 nmol/L, with patients suffering from headaches having the lowest levels [11]. A general improvement in terms of headaches was described after a daily cholecalciferol treatment of 1000 to 1500 IU [11]. Heath reported an even higher percentage (>90%) of patients with non–specific musculoskeletal pain to be found vitamin D deficient [6].

The European Male Aging Study (EMAS) from 2003 to 2005, investigating the association of low vitamin D and chronic widespread pain in men aged 40 to 79 years, revealed that participants with vitamin D levels of <15.6 ng/mL (lowest quintile) had a higher new onset of chronic widespread pain than patients from the upper quintile. In addition, they showed a significantly increased BMI and a clearly higher risk for depression, so that no independent association between chronic pain and the 25(OH)D level in men could be found [74].

## 6. Chronic Diseases as Risk Factor for Vitamin D Deficiency and Reduced Bone Strength (Secondary Osteoporosis)

Considering the classical actions of vitamin D, it is self-evident that decreased 25(OH)D levels constitute a major risk factor for developing systemic bone diseases such as hepatic osteodystrophy or diabetic osteopathy [17,27,60,75,76,77,78,79,80,81,82]. Table 1 summarizes several studies regarding the effect of chronic diseases on 25(OH)D status and bone mineral density. In patients with chronic liver diseases, 25(OH)D deficiency is associated with decreased gene expression levels of CYP2R1, CYP27A1 and GC (vitamin D-binding protein) as well as increased DHCR7 (7-dehydrocholesterol reductase) gene expression levels in the liver which positively correlates with a decrease in bone mineral density [80,82,83]. Furthermore, activation of 25(OH)D to 1,25(OH)D in the kidney is hampered by diseases affecting liver function, e.g., progressed diabetes mellitus and chronic kidney diseases [84]. Interestingly, there is a positive correlation between vitamin D status and diabetes mellitus already at the time of diagnosis [85]. 

Already in children, hepatic osteodystrophy or diabetic osteopathy comprises vitamin D deficiency rickets, low bone mass, and fractures caused by malnutrition and malabsorption. Here, aggressive treatment with ergocalciferol or cholecalciferol is required [86]. Interestingly, while initial studies suggested that parental vitamin D therapy may delay the development of hepatic osteodystrophy and diabetic osteopathy, more recent studies showed no significant improvement of BMD in these patients despite improved 25(OH)D serum levels [87,88,89]. Only a combination of oral vitamin D and bisphosphonates improved BMD in liver patients [90,91,92,93]. 

## 7. Conclusions

Vitamin D, the sunshine vitamin, has a key role in health and quality of life. While in the past it was insufficiently described as an “unknown ingredient” curing epidemic diseases, it is now acknowledged to be a “hormone” influencing metabolic pathways via its own receptors Vitamin D Substitution. 

Vitamin D is supplied by cutaneous synthesis depending, among other things, on sunlight exposure and dietary intake. Today, our food hardly contains the necessary quantities of essential vitamins, thereby necessitating supplementations. Consequently, vitamin D deficiency is quite common and can lead to severe diseases and even death. Type and severity of the symptoms may differ substantially, depending on the underlying disease. Rickets, occurring already in early childhood, and osteomalacia, a reduced bone mineralization primarily found in adults, can be completely cured if detected correctly and treated appropriately. Osteoporosis is commonly seen in older people and leads to an elevated risk of osteoporotic fractures, which are associated with a high morbidity and mortality. 

It was attempted, with partial success, to show that a vitamin D deficiency can also cause severe musculoskeletal pain. Many patients with a long history of non-specific back pain and multiple clinical investigations without any clear diagnosis had been found vitamin D deficient. An improvement of symptoms could be achieved by vitamin D substitution. 

It is therefore important to take into consideration a potential vitamin D deficiency when treating patients with non-specific musculoskeletal pain, muscle weakness, a reduced postural control as well as fractures after low-energy trauma.

According to the literature, a 25(OH)D level of >30 ng/mL prevents severe diseases and should be achieved. With daily doses of vitamin D below 600 IU, apparently no positive effect regarding a relief of musculoskeletal pain or prevention of osteoporotic fractures had been found. This clearly implies that further research is still needed to optimize vitamin D supplementation dosings and define recommendation guidelines regarding bone health, muscle functioning in acute situations as well as rehabilitation.

## Figures and Tables

**Table 1:** Prevalence and degree of bone disease related to chronic diseases of various etiologies.

Reference	*n*	Etiology	Prevalence of	
			Osteopenia	Osteoporosis
Spencer *et al.*, 1986 [94]	96	alcoholic liver disease	*n.s.*	47%
Diamond *et al.*, 1989 [95]	22	hemochromatosis	*n.s.*	45%
Diamond *et al.*, 1989 [96]	80	mixed etiology	*n.s.*	21%
Diamond *et al.*, 1989 [97]	28	alcoholic liver disease	28%	38%
Diamond *et al.*, 1990 [98]	115	mixed etiology	*n.s.*	28%–75%
Guañabens *et al.*, 1990 [99]	20	primary biliary cirrhosis	35%	
Gonzalez-Calvin *et al.*, 1993 [100]	39	alcoholic liver disease	23%	*n.s.*
Kayath *et al.*, 1994 [101]	90	insulin-dependent diabetes mellitus	34%	
Lindor *et al.*, 1995 [102]	88	primary biliary cirrhosis	*n.s.*	35%
Monegal *et al.*, 1997 [103]	58	mixed etiology (cirrhosis)	*n.s.*	43%
Sinigaglia *et al.*, 1997 [104]	32	hemochromatosis	*n.s.*	28%
Kayath *et al.*, 1998 [105]	23	insulin-dependent diabetes mellitus	48%	*n.s.*
Gunczler *et al.*, 1998 [106]	26	insulin-dependent diabetes mellitus	92.6%	
Angulo *et al.*, 1998 [107]	81	primary sclerosing cholangitis	*n.s.*	17%
Gallegy–Rojo *et al.*, 1998 [108]	32	viral cirrhosis	*n.s.*	53%
Pollak *et al.*, 1998 [109]	63	inflammatory bowel disease	42%	41%
Kemink *et al.*, 2000 [110]	35	insulin–dependent diabetes mellitus	62%	-
Ardizzone *et al.*, 2000 [111]	51	Crohn’s disease	55%	37%
	40	ulcerative colitis	76%	18%
Duarte *et al.*, 2001 [112]	100	viral cirrhosis	*n.s.*	25%
Menon *et al.*, 2001 [113]	176	primary biliary cirrhosis	*n.s.*	20%
Kim *et al.*, 2003 [114]	19	alcoholic liver disease	50%	22%
Sokhi *et al.*, 2004 [115]	104	mixed etiology (cirrhosis)	34.6%	11.5%
Jahnsen *et al.*, 2004 [116]	60	Crohn’s disease	22%	
	60	ulcerative colitis	27%	
Floreani *et al.*, 2005 [117]	35	primary biliary cirrhosis	*n.s.*	14.2%
	49	viral cirrhosis	*n.s.*	14.3%
Auletta *et al.*, 2005 [118]	30	chronic hepatitis	44%	20%
Guggenbuhl *et al.*, 2005 [119]	38	hemochromatosis	44.7%	34.2%
Zali *et al.*, 2006 [120]	165	inflammatory bowel disease	26.7%	5.4%
Hofmann *et al.*, 2008 [121]	30	chronic hepatitis C	43%	13%
Mounach *et al.*, 2008 [76]	33	primary biliary cirrhosis	*n.s.*	51.5%
Lumachi *et al.*, 2009 [122]	18	insulin–dependent diabetes mellitus	61.1%	*n.s.*
Malik *et al.*, 2009 [123]	57	alcoholic liver disease	17.5%	
George *et al.*, 2009 [17]	72	viral and alcoholic (cirrhosis)	68%	
Goral *et al.*, 2010 [124]	55	mixed etiology (cirrhosis)	*n.s.*	37%
Loria *et al.*, 2010 [125]	35	mixed etiology (cirrhosis)	26%	14%
Wariaghli *et al.*, 2010 [126]	64	mixed etiology (cirrhosis)	*n.s.*	45.3%
Wibaux *et al.*, 2011 [127]	99	mixed etiology (cirrhosis)	35%	38%
Angulo *et al.*, 2011 [128]	237	primary sclerosing cholangitis	*n.s.*	15%–75%
Choudhary *et al.*, 2011 [21]	115	viral and alcoholic (cirrhosis)	93.7%–97%	
Alcalde Vargas *et al.*, 2012 [129]	486	mixed etiology (cirrhosis)	22%–43%	4%–23%
Pardee *et al.*, 2012 [130]	38	non–alcoholic fatty liver disease	45%	
Leidig *et al.*, 2014 [131]	139	diabetes mellitus type 1	*n.s.*	27%
	243	diabetes mellitus type 2	*n.s.*	14%
Mathen *et al.*, 2015 [132]	150	diabetes mellitus type 2	32%	35%
Chinnaratha *et al.*, 2015 [133]	406	mixed etiology (cirrhosis)	46%	21%

*n* = number of patients included; n.s. = not specified.

## Abbreviations

The following abbreviations are used in this manuscript:

1,25(OH)D	1,25-dihydroxyvitamin D
1-OHase	25-hydroxyvitamn D-1-hydroxylase
24OHase	enzyme 25-hydroxyvitamin D_3_-24-hydroxylase
25(OH)D	25-dihydroxyvitamin D
7-DHC	7-Dehydrocholesterol
BMD	bone mineral density
DBP	vitamin D-binding protein
DHCR7	7-Dehydrocholesterol-reductase
DPYD	deoxypyridinoline
DXA	dual-energy X-ray absorptiometry
FGF-3	fibroblast growth factors
PTH	parathyroid hormone
RANK	receptor activator of NFkB
RANKL	receptor activator of NFKB ligand
RXR	retinoid × receptor
UV	ultraviolet
VDR	vitamin D receptor
VDREs	vitamin D response elements

## References

[1] Funk C. (1911). On the chemical nature of the substance which cures polyneuritis in birds induced by a diet of polished rice. J. Physiol..

[2] Lund J., Deluca H.F. (1966). Biologically active metabolite of vitamin D_3_ from bone liver and blood serum. J. Lipid. Res..

[3] Deluca H.F. (2014). History of the discovery of vitamin D and its active metabolites. Bonekey Rep..

[4] Wacker M., Holick M.F. (2013). Vitamin D—Effects on skeletal and extraskeletal health and the need for supplementation. Nutrients.

[5] Gendelman O., Itzhaki D., Makarov S., Bennun M., Amital H. (2015). A randomized double-blind placebo-controlled study adding high dose vitamin D to analgesic regimens in patients with musculoskeletal pain. Lupus.

[6] Heath K.M., Elovic E.P. (2006). Vitamin D deficiency—Implications in the rehabilitation setting. Am. J. Phys. Med. Rehab..

[7] Shinchuk L., Holick M.F. (2007). Vitamin D and rehabilitation: Improving functional outcomes. Nutr. Clin. Pract..

[8] Munns C.F., Shaw N., Kiely M., Specker B.L., Thacher T.D., Ozono K., Michigami T., Tiosano D., Mughal M.Z., Makitie O. (2016). Global consensus recommendations on prevention and management of nutritional rickets. J. Clin. Endocrinol. Metab..

[9] Zhang R.H., He D.H., Zhou B., Zhu Y.B., Zhao D., Huang L.C., Ding G.Q. (2015). Analysis of vitamin D status in men highly exposed to sunlight. Biomed. Environ. Sci..

[10] Holick M.F. (2011). Vitamin D: A d-lightful solution for health. J. Invest. Med..

[11] Knutsen K.V., Brekke M., Gjelstad S., Lagerlov P. (2010). Vitamin D status in patients with musculoskeletal pain, fatigue and headache: A cross-sectional descriptive study in a multi-ethnic general practice in Norway. Scand. J. Prim. Health.

[12] Reuss-Borst M.A. (2014). Metabolic bone disease osteomalacia. Z. Rheumatol..

[13] Barvencik F., Amling M. (2015). Vitamin D metabolism of the bone. Orthopade.

[14] Mutt S.J., Hypponen E., Saarnio J., Jarvelin M.R., Herzig K.H. (2014). Vitamin D and adipose tissue—More than storage. Front. Physiol..

[15] Sutton A.L.M., MacDonald P.N. (2003). Vitamin D: More than a "bone-a-fide" hormone. Mol. Endocrinol..

[16] Ritter C.S., Brown A.J. (2011). Direct suppression of Pth gene expression by the vitamin D prohormones doxercalciferol and calcidiol requires the vitamin D receptor. J. Mol. Endocrinol..

[17] George J., Ganesh H.K., Acharya S., Bandgar T.R., Shivane V., Karvat A., Bhatia S.J., Shah S., Menon P.S., Shah N. (2009). Bone mineral density and disorders of mineral metabolism in chronic liver disease. World J. Gastroenterol..

[18] Lavi-Moshayoff V., Wasserman G., Meir T., Silver J., Naveh-Many T. (2010). Pth increases fgf23 gene expression and mediates the high–fgf23 levels of experimental kidney failure: A bone parathyroid feedback loop. Am. J. Physiol. Renal..

[19] Christensen M.H., Apalset E.M., Nordbo Y., Varhaug J.E., Mellgren G., Lien E.A. (2013). 1,25-dihydroxyvitamin D and the vitamin D receptor gene polymorphism apa1 influence bone mineral density in primary hyperparathyroidism. PLoS ONE.

[20] Tejwani V., Qian Q. (2013). Calcium regulation and bone mineral metabolism in elderly patients with chronic kidney disease. Nutrients.

[21] Choudhary N.S., Tomar M., Chawla Y.K., Bhadada S.K., Khandelwal N., Dhiman R.K., Duseja A., Bhansali A. (2011). Hepatic osteodystrophy is common in patients with noncholestatic liver disease. Dig. Dis. Sci..

[22] Servier Medical Art Powerpoint Image Bank. http://www.servier.com/Powerpoint–image-bank.

[23] Ceglia L. (2009). Vitamin D and its role in skeletal muscle. Curr. Opin. Clin. Nutr..

[24] Umhau J.C., George D.T., Heaney R.P., Lewis M.D., Ursano R.J., Heilig M., Hibbeln J.R., Schwandt M.L. (2013). Low vitamin D status and suicide: A case-control study of active duty military service members. PLoS ONE.

[25] Takahashi N., Udagawa N., Suda T. (2014). Vitamin D endocrine system and osteoclasts. Bonekey Rep..

[26] Carey R.M., Siragy H.M. (2003). The intrarenal renin-angiotensin system and diabetic nephropathy. Trends Endocrinol. Met..

[27] Williams S., Malatesta K., Norris K. (2009). Vitamin D and chronic kidney disease. Ethn. Dis..

[28] Putz-Bankuti C., Pilz S., Stojakovic T., Scharnagl H., Pieber T.R., Trauner M., Obermayer-Pietsch B., Stauber R.E. (2012). Association of 25-hydroxyvitamin D levels with liver dysfunction and mortality in chronic liver disease. Liver Int..

[29] Li Y.C. (2010). Renoprotective effects of vitamin D analogs. Kidney Int..

[30] Callegari E.T., Reavley N., Garland S.M., Gorelik A., Wark J.D., Team S.-D.S. (2015). Vitamin D status, bone mineral density and mental health in young Australian women: The safe-D study. J. Public Health Res..

[31] Huang J.Y., Arnold D., Qiu C.F., Miller R.S., Williams M.A., Enquobahrie D.A. (2014). Association of serum vitamin D with symptoms of depression and anxiety in early pregnancy. J. Womens Health.

[32] Shuler F.D., Wingate M.K., Moore G.H., Giangarra C. (2012). Sports health benefits of vitamin D. Sports Health.

[33] Masood T., Kushwaha R.S., Singh R., Sailwal S., Pandey H., Varma A., Singh R.K., Cornelissen G. (2015). Circadian rhythm of serum 25 (OH) vitamin D, calcium and phosphorus levels in the treatment and management of type-2 diabetic patients. Drug Discov. Ther..

[34] Looker A.C., Johnson C.L., Lacher D.A., Pfeiffer C.M., Schleicher R.L., Sempos C.T. (2011). Vitamin D status: United States, 2001–2006. NCHS Data Brief.

[35] Rabenberg M., Scheidt-Nave C., Busch M.A., Rieckmann N., Hintzpeter B., Mensink G.B.M. (2015). Vitamin D status among adults in Germany—Results from the German health interview and examination survey for adults (DEGS1). BMC Public Health.

[36] Amling M. (2015). Calcium and vitamin D in bone metabolism. Clinical importance for fracture treatment. Unfallchirurg.

[37] Gilchrest B.A. (2008). Sun exposure and vitamin D sufficiency. Am. J. Clin. Nutr..

[38] El-Sonbaty M.R., Abdul-Ghaffar N.U. (1996). Vitamin D deficiency in veiled Kuwaiti women. Eur. J. Clin. Nutr..

[39] Diamond T.H., Levy S., Smith A., Day P. (2002). High bone turnover in muslim women with vitamin D deficiency. Med. J. Aust..

[40] Allali F., el Aichaoui S., Saoud B., Maaroufi H., Abouqal R., Hajjaj-Hassouni N. (2006). The impact of clothing style on bone mineral density among post menopausal women in Morocco: A case–control study. BMC Public Health.

[41] Metcalfe D. (2008). The pathophysiology of osteoporotic hip fracture. Mcgill J. Med..

[42] Rader C.P., Corsten N., Rolf O. (2015). Osteomalacia and vitamin D deficiency. Orthopade.

[43] Bischoff-Ferrari H.A., Dawson-Hughes B., Staehelin H.B., Orav J.E., Stuck A.E., Theiler R., Wong J.B., Egli A., Kiel D.P., Henschkowski J. (2009). Fall prevention with supplemental and active forms of vitamin D: A meta-analysis of randomised controlled trials. Br. Med. J..

[44] Vernazza P., Stoliaroff A., Boggian K., Schlegel M. Vitamin D Substitution. https://www.guidelines.ch/page/pdf/53.

[45] DVO Leitlinie Osteoporose 2014 Kurzfassung und Langfassung. http://www.dv–osteologie.org/uploads/Leitlinie%202014/DVO–Leitlinie%20Osteoporose%202014%20Kurzfassung%20und%20Langfassung%20Version%201a%2012%2001%202016.pdf.

[46] Gesundes Altern: Höhere Vitamin–D Dosis Kann Sturzrisiko Erhöhen. http://www.aerzteblatt.de/nachrichten/65299/Gesundes–Altern–Hoehere–Vitamin–D–Dosis–kann–Sturzrisiko–erhoehen.

[47] Sharifi N., Amani R., Hajiani E., Cheraghian B. (2016). Women may respond different from men to vitamin D supplementation regarding cardiometabolic biomarkers. Exp. Biol. Med..

[48] O’Riordan J.L.H., Bijvoet O.L.M. (2014). Rickets before the discovery of vitamin D. Bone Key Rep..

[49] Bivins R. (2014). Ideology and disease identity: The politics of rickets, 1929–1982. Med. Humanit..

[50] Dunn P.M. (2005). Sir robert hutchison (1871–1960) of London and the causes and treatment of rickets. Arch. Dis. Child. Fetal.

[51] Goldacre M., Hall N., Yeates D.G.R. (2014). Hospitalisation for children with rickets in England: A historical perspective. Lancet.

[52] Fukumoto S., Ozono K., Michigami T., Minagawa M., Okazaki R., Sugimoto T., Takeuchi Y., Matsumoto T. (2015). Pathogenesis and diagnostic criteria for rickets and osteomalacia-proposal by an expert panel supported by the ministry of health, labour and welfare, Japan, the Japanese society for bone and mineral research, and the Japan endocrine society. J. Bone Miner. Metab..

[53] Lopresti M., Daolio P.A., Rancati J.M., Ligabue N., Andreolli A., Panella L. (2015). Rehabilitation of a patient receiving a large–resection hip prosthesis because of a phosphaturic mesenchymal tumor. Clin. Pract..

[54] Arantes H.P., Gimeno S.G., Chiang A.Y., Bilezikian J.P., Lazaretti-Castro M. (2016). Incidence of vertebral fractures in calcium and vitamin D–supplemented postmenopausal Brazilian women with osteopenia or osteoporosis: Data from arzoxifene generations trial. Arch. Endocrinol. Metab..

[55] Siris E., Adler R., Bilezikian J., Bolognese M., Dawson-Hughes B., Favus M., Harris S., Jan de Beur S., Khosla S., Lane N. (2014). The clinical diagnosis of osteoporosis: A position statement from the national bone health alliance working group. Osteoporo. Int..

[56] Yoo J.H., Moon S.H., Ha Y.C., Lee D.Y., Gong H.S., Park S.Y., Yang K.H. (2015). Osteoporotic fracture: 2015 position statement of the Korean society for bone and mineral research. J. Bone Metab..

[57] Shane E., Burr D., Ebeling P.R., Abrahamsen B., Adler R.A., Brown T.D., Cheung A.M., Cosman F., Curtis J.R., Dell R. (2010). Atypical subtrochanteric and diaphyseal femoral fractures: Report of a task force of the American society for bone and mineral research. J. Bone Miner. Res..

[58] Jackson R.D., LaCroix A.Z., Gass M., Wallace R.B., Robbins J., Lewis C.E., Bassford T., Beresford S.A., Black H.R., Blanchette P. (2006). Calcium plus vitamin D supplementation and the risk of fractures. N. Engl. J. Med..

[59] Johnell O., Kanis J.A. (2006). An estimate of the worldwide prevalence and disability associated with osteoporotic fractures. Osteoporosis. Int..

[60] Rode A., Fourlanos S., Nicoll A. (2010). Oral vitamin D replacement is effective in chronic liver disease. Gastroenterol. Clin. Biol..

[61] Robert-Koch-Institut (2009). Prävalenz von Osteoporose.

[62] Servier Medical Art Bone Structure. http://www.servier.com/slidekit/?item=2.

[63] Winzenberg T., van der Mei I., Mason R.S., Nowson C., Jones G. (2012). Vitamin D and the musculoskeletal health of older adults. Aust. Fam. Physician.

[64] Bischoff-Ferrari H.A., Willett W.C., Orav E.J., Lips P., Meunier P.J., Lyons R.A., Flicker L., Wark J., Jackson R.D., Cauley J.A. (2012). A pooled analysis of vitamin D dose requirements for fracture prevention. N. Engl. J. Med..

[65] Bischoff-Ferrari H.A., Dawson-Hughes B., Baron J.A., Burckhardt P., Li R., Spiegelman D., Specker B., Orav J.E., Wong J.B., Staehelin H.B. (2007). Calcium intake and hip fracture risk in men and women: A meta-analysis of prospective cohort studies and randomized controlled trials. Am. J. Clin. Nutr..

[66] Cummings S.R., Kiel D.P., Black D.M. (2016). Vitamin D supplementation and increased risk of falling: A cautionary tale of vitamin supplements retold. JAMA Intern. Med..

[67] Huo Y.R., Suriyaarachchi P., Gomez F., Curcio C.L., Boersma D., Muir S.W., Montero-Odasso M., Gunawardene P., Demontiero O., Duque G. (2015). Phenotype of osteosarcopenia in older individuals with a history of falling. J. Am. Med. Dir. Assoc..

[68] Kim M.K., Baek K.H., Song K.H., Kang M., Park C.Y., Lee W.Y., Oh K.W. (2011). Vitamin D deficiency is associated with sarcopenia in older Koreans, regardless of obesity: The fourth Korea national health and nutrition examination surveys (KNHANES IV) 2009. J. Clin. Endocr. Metab..

[69] Snijder M.B., van Schoor N.M., Pluijm S.M.F., van Dam R.M., Visser M., Lips P. (2006). Vitamin D status in relation to one–year risk of recurrent falling in older men and women. J. Clin. Endocr. Metab..

[70] Pike J.W. (2016). Closing in on vitamin D action in skeletal muscle: Early activity in muscle stem cells?. Endocrinology.

[71] Campbell W.W., Johnson C.A., McCabe G.P., Carnell N.S. (2008). Dietary protein requirements of younger and older adults. Am. J. Clin. Nutr..

[72] De Koning E.J., van Schoor N.M., Penninx B.W.J.H., Elders P.J.M., Heijboer A.C., Smit J.H., Bet P.M., van Tulder M.W., den Heijer M., van Marwijk H.W.J. (2015). Vitamin D supplementation to prevent depression and poor physical function in older adults: Study protocol of the D-vitaal study, a randomized placebo-controlled clinical trial. BMC Geriatr..

[73] Bischoff-Ferrari H.A., Dawson-Hughes B., Orav E.J., Staehelin H.B., Meyer O.W., Theiler R., Dick W., Willett W.C., Egli A. (2016). Monthly high-dose vitamin D treatment for the prevention of functional decline: A randomized clinical trial. JAMA Intern. Med..

[74] McCabe P.S., Pye S.R., Mc Beth J., Lee D.M., Tajar A., Bartfai G., Boonen S., Bouillon R., Casanueva F., Finn J.D. (2016). Low vitamin D and the risk of developing chronic widespread pain: Results from the european male ageing study. BMC Musculoskelet. Dis..

[75] Compston J.E. (1986). Hepatic osteodystrophy—Vitamin-D metabolism in patients with liver–disease. Gut.

[76] Mounach A., Ouzzif Z., Wariaghli G., Achemlal L., Benbaghdadi I., Aouragh A., Bezza A., el Maghraoui A. (2008). Primary biliary cirrhosis and osteoporosis: A case–control study. J. Bone Miner. Metab..

[77] Arteh J., Narra S., Nair S. (2010). Prevalence of vitamin D deficiency in chronic liver disease. Digest. Dis. Sci..

[78] Xiao X., Wang Y., Hou Y., Han F., Ren J., Hu Z. (2016). Vitamin D deficiency and related risk factors in patients with diabetic nephropathy. J. Int. Med. Res..

[79] Ehnert S., Freude T., Ihle C., Mayer L., Braun B., Graeser J., Flesch I., Stockle U., Nussler A.K., Pscherer S. (2015). Factors circulating in the blood of type 2 diabetes mellitus patients affect osteoblast maturation—Description of a novel *in vitro* model. Exp. Cell Res..

[80] Nussler A.K., Wildemann B., Freude T., Litzka C., Soldo P., Friess H., Hammad S., Hengstler J.G., Braun K.F., Trak-Smayra V. (2014). Chronic CCl4 intoxication causes liver and bone damage similar to the human pathology of hepatic osteodystrophy: A mouse model to analyse the liver-bone axis. Arch. Toxicol..

[81] Haug A.T., Braun K.F., Ehnert S., Mayer L., Stockle U., Nussler A.K., Pscherer S., Freude T. (2014). Gene expression changes in cancellous bone of type 2 diabetics: A biomolecular basis for diabetic bone disease. Langenbeck Arch. Surg..

[82] Hochrath K., Ehnert S., Ackert-Bicknell C.L., Lau Y., Schmid A., Krawczyk M., Hengstler J.G., Dunn J., Hiththetiya K., Rathkolb B. (2013). Modeling hepatic osteodystrophy in Abcb4 deficient mice. Bone.

[83] Bu F.X., Armas L., Lappe J., Zhou Y., Gao G.M., Wang H.W., Recker R., Zhao L.J. (2010). Comprehensive association analysis of nine candidate genes with serum 25-hydroxy vitamin D levels among healthy caucasian subjects. Hum. Genet..

[84] Jones G. (2007). Expanding role for vitamin D in chronic kidney disease: Importance of blood 25-OH-D levels and extra-renal 1 alpha-hydroxylase in the classical and nonclassical actions of 1 alpha,25-dihydroxyvitamin D-3. Semin. Dial..

[85] Vujosevic S., Borozan S., Radojevic N., Aligrudic S., Bozovic D. (2014). Relationship between 25-hydroxyvitamin D and newly diagnosed type 2 diabetes mellitus in postmenopausal women with osteoporosis. Med. Princ. Pract..

[86] Hogler W., Baumann U., Kelly D. (2012). Endocrine and bone metabolic complications in chronic liver disease and after liver transplantation in children. J. Pediatr. Gastr. Nutr..

[87] Maalouf N.M., Sakhaee K. (2006). Treatment of osteoporosis in patients with chronic liver disease and in liver transplant recipients. Curr. Treat. Options Gastroenterol..

[88] Yurci A., Kalkan A.O., Ozbakir O., Karaman A., Torun E., Kula M., Baskol M., Gursoy S., Yucesoy M., Bayram F. (2011). Efficacy of different therapeutic regimens on hepatic osteodystrophy in chronic viral liver disease. Eur. J. Gastroenterol. Hepatol..

[89] Neyestani T.R., Nikooyeh B., Kalayi A., Zahedirad M., Shariatzadeh N. (2015). A vitamin D-calcium-fortified yogurt drink decreased serum PTH but did not affect osteocalcin in subjects with type 2 diabetes. Int. J. Vitam. Nutr. Res..

[90] Wariaghli G., Allali F., el Maghraoui A., Hajjaj-Hassouni N. (2010). Osteoporosis in patients with primary biliary cirrhosis. Eur. J. Gastroenterol. Hepatol..

[91] Guanabens N., Pares A. (2011). Management of osteoporosis in liver disease. Clin. Res. Hepatol. Gastroenterol..

[92] Rudic J.S., Giljaca V., Krstic M.N., Bjelakovic G., Gluud C. (2011). Bisphosphonates for osteoporosis in primary biliary cirrhosis. Cochrane Database Syst. Rev..

[93] Luxon B.A. (2011). Bone disorders in chronic liver diseases. Curr. Gastroenterol. Rep..

[94] Spencer H., Rubio N., Rubio E., Indreika M., Seitam A. (1986). Chronic alcoholism. Frequently overlooked cause of osteoporosis in men. Am. J. Med..

[95] Diamond T., Stiel D., Posen S. (1989). Osteoporosis in hemochromatosis: Iron excess, gonadal deficiency, or other factors?. Ann. Intern. Med..

[96] Diamond T.H., Stiel D., Lunzer M., McDowall D., Eckstein R.P., Posen S. (1989). Hepatic osteodystrophy. Static and dynamic bone histomorphometry and serum bone Gla–protein in 80 patients with chronic liver disease. Gastroenterology.

[97] Diamond T., Stiel D., Lunzer M., Wilkinson M., Posen S. (1989). Ethanol reduces bone formation and may cause osteoporosis. Am. J. Med..

[98] Diamond T., Stiel D., Lunzer M., Wilkinson M., Roche J., Posen S. (1990). Osteoporosis and skeletal fractures in chronic liver disease. Gut.

[99] Guanabens N., Pares A., Marinoso L., Brancos M.A., Piera C., Serrano S., Rivera F., Rodes J. (1990). Factors influencing the development of metabolic bone disease in primary biliary cirrhosis. Am. J. Gastroenterol..

[100] Gonzalez-Calvin J.L., Garcia-Sanchez A., Bellot V., Munoz-Torres M., Raya-Alvarez E., Salvatierra-Rios D. (1993). Mineral metabolism, osteoblastic function and bone mass in chronic alcoholism. Alcohol Alcohol..

[101] Kayath M.J., Dib S.A., Vieira J.G.H. (1994). Prevalence and magnitude of osteopenia associated with insulin- dependent diabetes-mellitus. J. Diabetes Complicat..

[102] Lindor K.D., Janes C.H., Crippin J.S., Jorgensen R.A., Dickson E.R. (1995). Bone disease in primary biliary cirrhosis: Does ursodeoxycholic acid make a difference?. Hepatology.

[103] Monegal A., Navasa M., Guanabens N., Peris P., Pons F., de Osaba M.J.M., Rimola A., Rodes J., Munoz-Gomez J. (1997). Osteoporosis and bone mineral metabolism disorders in cirrhotic patients referred for orthotopic liver transplantation. Calcif. Tissue Int..

[104] Sinigaglia L., Fargion S., Fracanzani A.L., Binelli L., Battafarano N., Varenna M., Piperno A., Fiorelli G. (1997). Bone and joint involvement in genetic hemochromatosis: Role of cirrhosis and iron overload. J. Rheumatol..

[105] Kayath M.J., Tavares E.F., Dib S.A., Vieira J.G.H. (1998). Prospective bone mineral density evaluation in patients with insulin-dependent diabetes mellitus. J. Diabetes Complicat..

[106] Gunczler P., Lanes R., Paz-Martinez V., Martinis R., Esaa S., Colmenares V., Weisinger J.R. (1998). Decreased lumbar spine bone mass and low bone turnover in children and adolescents with insulin dependent diabetes mellitus followed longitudinally. J. Pediatr. Endocr. Met..

[107] Angulo P., Therneau T.M., Jorgensen A., DeSotel C.K., Egan K.S., Dickson E.R., Hay J.E., Lindor K.D. (1998). Bone disease in patients with primary sclerosing cholangitis: Prevalence, severity and prediction of progression. J. Hepatol..

[108] Gallego-Rojo F.J., Gonzalez-Calvin J.L., Munoz-Torres M., Mundi J.L., Fernandez-Perez R., Rodrigo-Moreno D. (1998). Bone mineral density, serum insulin-like growth factor I, and bone turnover markers in viral cirrhosis. Hepatology.

[109] Pollak R.D., Karmeli F., Eliakim R., Ackerman Z., Tabb K., Rachmilewitz D. (1998). Femoral neck osteopenia in patients with inflammatory bowel disease. Am. J. Gastroenterol..

[110] Kemink S.A., Hermus A.R., Swinkels L.M., Lutterman J.A., Smals A.G. (2000). Osteopenia in insulin-dependent diabetes mellitus; prevalence and aspects of pathophysiology. J. Endocrinol. Investig..

[111] Ardizzone S., Bollani S., Bettica P., Bevilacqua M., Molteni P., Porro G.B. (2000). Altered bone metabolism in inflammatory bowel disease: There is a difference between crohn’s disease and ulcerative colitis. J. Intern. Med..

[112] Duarte M.P., Farias M.L., Coelho H.S., Mendonca L.M., Stabnov L.M., do Carmo d Oliveira M., Lamy R.A., Oliveira D.S. (2001). Calcium-parathyroid hormone-vitamin D axis and metabolic bone disease in chronic viral liver disease. J. Gastroenterol. Hepatol..

[113] Menon K.V., Angulo P., Weston S., Dickson E.R., Lindor K.D. (2001). Bone disease in primary biliary cirrhosis: Independent indicators and rate of progression. J. Hepatol..

[114] Kim S., Koga T., Isobe M., Kern B.E., Yokochi T., Chin Y.E., Karsenty G., Taniguchi T., Takayanagi H. (2003). Stat1 functions as a cytoplasmic attenuator of runx2 in the transcriptional program of osteoblast differentiation. Genes Dev..

[115] Sokhi R.P., Anantharaju A., Kondaveeti R., Creech S.D., Islam K.K., van Thiel D.H. (2004). Bone mineral density among cirrhotic patients awaiting liver transplantation. Liver Transpl..

[116] Jahnsen J., Falch J.A., Mowinckel P., Aadland E. (2004). Bone mineral density in patients with inflammatory bowel disease: A population-based prospective two-year follow-up study. Scand. J. Gastroenterol..

[117] Floreani A., Mega A., Camozzi V., Baldo V., Plebani M., Burra P., Luisetto G. (2005). Is osteoporosis a peculiar association with primary biliary cirrhosis?. World J. Gastroenterol..

[118] Auletta M., Nuzzo V., Esposito A., Antonello S., Lupoli G., Federico F., de Puente A. (2005). Osteoporosis in men: A study in patients affected by chronic non-advanced liver disease. Clin. Cases Miner. Bone Metab..

[119] Guggenbuhl P., Deugnier Y., Boisdet J.F., Rolland Y., Perdriger A., Pawlotsky Y., Chales G. (2005). Bone mineral density in men with genetic hemochromatosis and HFE gene mutation. Osteoporos Int..

[120] Zali M., Bahari A., Firouzi F., Daryani N.E., Aghazadeh R., Emam M.M., Rezaie A., Shalmani H.M., Naderi N., Maleki B. (2006). Bone mineral density in Iranian patients with inflammatory bowel disease. Int. J. Colorectal Dis..

[121] Hofmann W.P., Kronenberger B., Bojunga J., Stamm B., Herrmann E., Bucker A., Mihm U., von Wagner M., Zeuzem S., Sarrazin C. (2008). Prospective study of bone mineral density and metabolism in patients with chronic hepatitis C during pegylated interferon alpha and ribavirin therapy. J. Viral Hepat..

[122] Lumachi F., Camozzi V., Tombolan V., Luisetto G. (2009). Bone mineral density, osteocalcin, and bone-specific alkaline phosphatase in patients with insulin-dependent diabetes mellitus. Ann. N. Y. Acad. Sci..

[123] Malik P., Gasser R.W., Kemmler G., Moncayo R., Finkenstedt G., Kurz M., Fleischhacker W.W. (2009). Low bone mineral density and impaired bone metabolism in young alcoholic patients without liver cirrhosis: A cross-sectional study. Alcohol. Clin. Exp. Res..

[124] Goral V., Simsek M., Mete N. (2010). Hepatic osteodystrophy and liver cirrhosis. World J. Gastroenterol..

[125] Loria I., Albanese C., Giusto M., Galtieri P.A., Giannelli V., Lucidi C., di Menna S., Pirazzi C., Corradini S.G., Mennini G. (2010). Bone disorders in patients with chronic liver disease awaiting liver transplantation. Transplant. Proc..

[126] Wariaghli G., Mounach A., Achemlal L., Benbaghdadi I., Aouragh A., Bezza A., el Maghraoui A. (2010). Osteoporosis in chronic liver disease: A case-control study. Rheumatol. Int..

[127] Wibaux C., Legroux-Gerot I., Dharancy S., Boleslawski E., Declerck N., Canva V., Mathurin P., Pruvot F.R., Cortet B. (2011). Assessing bone status in patients awaiting liver transplantation. Jt. Bone Spine.

[128] Angulo P., Grandison G.A., Fong D.G., Keach J.C., Lindor K.D., Bjornsson E., Koch A. (2011). Bone disease in patients with primary sclerosing cholangitis. Gastroenterology.

[129] Vargas A.A., Pascasio Acevedo J.M., Domingo I.G., Jimenez R.G., Martin J.M.S., Rios M.T.F., Mota M.S., Gallego A.G., Bravo M.A.G. (2012). Prevalence and characteristics of bone disease in cirrhotic patients under evaluation for liver transplantation. Transplant. Proc..

[130] Pardee P.E., Dunn W., Schwimmer J.B. (2012). Non-alcoholic fatty liver disease is associated with low bone mineral density in obese children. Aliment. Pharmacol. Ther..

[131] Bruckner G.L., Grobholz S., Bruckner T., Scheidt-Nave C., Nawroth P., Schneider J.G. (2014). Prevalence and determinants of osteoporosis in patients with type 1 and type 2 diabetes mellitus. BMC Endocr. Disord..

[132] Mathen P.G., Thabah M.M., Zachariah B., Das A.K. (2015). Decreased bone mineral density at the femoral neck and lumbar spine in south Indian patients with type 2 diabetes. J. Clin. Diagn. Res..

[133] Chinnaratha M.A., Chaudhary S., Doogue M., McCormick R.J., Woodman R.J., Wigg A.J. (2015). Prevalence of hepatic osteodystrophy and vitamin D deficiency in cirrhosis. Intern. Med. J..

